# Modeling the Growth and Death of *Staphylococcus aureus* against *Melaleuca armillaris* Essential Oil at Different pH Conditions

**DOI:** 10.3390/antibiotics10020222

**Published:** 2021-02-23

**Authors:** Daniel Buldain, Lihuel Gortari Castillo, María Laura Marchetti, Karen Julca Lozano, Arnaldo Bandoni, Nora Mestorino

**Affiliations:** 1 Laboratorio de Estudios Farmacológicos y Toxicológicos (LEFyT), Facultad de Ciencias Veterinarias, Universidad Nacional de La Plata, La Plata B1900, Argentina; lgortari@fcv.unlp.edu.ar (L.G.C.); mlmarchetti@fcv.unlp.edu.ar (M.L.M.); k.julcalozano@gmail.com (K.J.L.); noram@fcv.unlp.edu.ar (N.M.); 2 Consejo Nacional de Investigaciones Científicas y Técnicas (CONICET), La Plata B1900, Argentina; 3 Facultad de Farmacia y Bioquímica, Cátedra de Farmacognosia, Universidad de Buenos Aires, C.A. de Buenos Aires C1113AAD, Argentina; abandoni@ffyb.uba.ar; 4 Instituto de Química y Metabolismo del Fármaco (IQUIMEFA), CONICET-Universidad de Buenos Aires, C.A. de Buenos Aires C1113AAD, Argentina

**Keywords:** *Staphylococcus aureus*, *Melaleuca armillaris*, essential oil, Gompertz model, Sigmoid model, antibacterial

## Abstract

Essential oils (EO) are a great antimicrobial resource against bacterial resistance in public health. Math models are useful in describing the growth, survival, and inactivation of microorganisms against antimicrobials. We evaluated the antimicrobial activity of *Melaleuca armillaris* EO obtained from plants placed in the province of Buenos Aires (Argentina) against *Staphylococcus aureus.* The minimum inhibitory and bactericidal concentrations were close and decreased, slightly acidifying the medium from pH 7.4 to 6.5 and 5.0. This result was also evidenced by applying a sigmoid model, where the time and EO concentration necessaries to achieve 50% of the maximum effect decreased when the medium was acidified. Moreover, at pH 7.4, applying the Gompertz model, we found that subinhibitory concentrations of EO decreased the growth rate and the maximum population density and increased the latency period concerning the control. Additionally, we established physicochemical parameters for quality control and standardization of *M. armillaris* EO. Mathematical modeling allowed us to estimate key parameters in the behavior of *S. aureus* and *Melaleuca armillaris* EO at different pH. This is interesting in situations where the pH changes are relevant, such as the control of intracellular infections in public health or the development of preservatives for the food industry.

## 1. Introduction

Antimicrobial resistance is a critical problem with a high prevalence in both human and animal medicine [1]. Essential oils (EO) are a great resource as an alternative therapy, providing a lot of antimicrobials (ATMs) compounds produced by aromatic plants. In addition to their usefulness in medicine, essential oils are of great importance in the food industry to guarantee food preservation and safety [2]. These can act as bacteriostatics or bactericides in several ways by responding to different action mechanisms and having a wide variety of target sites, which generally lead to destabilization of the phospholipid bilayer, destruction of the function and composition of the plasma membrane, loss of vital intracellular components, and inactivation of enzyme mechanisms [3]. The genus *Melaleuca* belongs to the Myrtaceae family, which contains a lot of species of plants producing EO. Among the species of *Melaleuca* genus, *Melaleuca armillaris* Sm. is one of the most widely cultivated. It is commonly known as Honey bracelet myrtle and grows as a small tree or as a large bush. Investigations by GC-MS (gas chromatography coupled to mass spectrometry) of its essential oil revealed the presence of 1.8 cineole as the main component [4,5,6,7]. Several authors evaluated the biological activities for this essential oil. For instance, Rizk et al. (2012) obtained good results in vivo using it for the treatment of the parasite *Schistosoma mansoni*, responding to the oxidative activity generated by this pathogen [8]. In vitro inhibitory activity was also found against *Staphylococcus aureus* [4] and other bacterial species such as *Bacillus subtilis*, *Staphylococcus epidermidis*, *Escherichia coli*, and *Pseudomonas aeruginosa* [9]. Reports about this plant EO are scarce, but these have potential as antimicrobials, and more studies must be conducted to exploit them. Antibacterial agents from plants can act as important sources of new antibiotics, efflux pump inhibitors, compounds that target bacterial virulence, or can be used in combination with existing drugs [10].

*S. aureus* is recognized worldwide as a causative pathogen of different types of infections in humans and animals. Moreover, it is commonly found in animals that are intended for food production such as dairy cows, sheep, and goats, particularly when they are affected by subclinical mastitis [11], and represent a high risk for human consumption [12]. This microorganism can express a wide spectrum of pathogenic factors used to colonize, invade, and infect the host [13]. This pathogen can survive intracellularly contributing to the recurrence of infections like mastitis in cows. Despite several ATMs showing good in vitro activity, the cure rates are low, because the bacteria do not fade to adequate concentrations and exposure times sufficient to eradicate them [14]. This may be related to the low intracellular penetration of some ATMs or their loss of activity at the acidic pH of lysosomes, the low diffusion of acidic ATMs through the lysosomal membrane due to their high ionization at neutral extracellular or cytoplasmic pH, and the poor retention of ATMs inside that enter freely [15]. Ideally, the ATMs needed to treat these infections should penetrate the phagocytic cells in adequate concentrations and time, not be metabolized in the cells, and be active at acidic pH [16]. 

The change of pH can influence the antimicrobial activity of the different molecules. For example, β-lactams increase their potency by acidifying the media [17], while macrolides lose their antibacterial activity with a decrease in pH [18]. This is interesting for the treatment of intracellular pathogens causing infection such as *S. aureus*, which can internalize in the phagolysosome where the pH is close to 5 [19]. The susceptibility of microorganisms to EO seemed to be higher at lower pH; the hydrophobicity of EO is higher at low pH, and this favors their dissolution in the lipids of the cell membrane [20].

There are different methods to evaluate antimicrobial activity. A microdilution in broth is the most common technique, standardized by the Clinical & Laboratory Standards Institute (CLSI). This provides very useful parameters like a minimum inhibitory concentration (MIC) and minimum bactericidal concentration (MBC). The problem with these parameters is that they give us static information. On the other hand, the time-kill assay is a very useful method that describes the antimicrobial activity of a compound dynamically, allowing the analysis of the bacterial behavior in the presence of antimicrobial along the time [21]. The curves (bacterial concentrations vs. time) obtained by this last kind of technic can be analyzed by mathematical models. Kinetic models can explain the behavior of a bacterial inoculum in the time because of the presence of an antimicrobial or the change of environmental variables [22]. There exist several kinetic models like Gompertz and square root models that can provide the growth rate (μ), lag period duration (LPD), and the maximum population density (MPD) [23]. There also exist models to describe the survival or the destruction of bacteria over the time, like the sigmoid minus the base model [24], which is similar to E_max_ models [25].

Modeling the antimicrobial activity of natural products like EO is mainly associated to the research of food preservatives [22]. There are no studies on models applied to natural products with antimicrobial activity in veterinary medicine. This is a powerful tool to understand the behavior of bacteria against new therapeutic alternatives molecules to control infections.

Our work aimed to describe by mathematical models the behavior of *S. aureus* against *M. armillaris* EO at different pH, emulating extra- and intracellular conditions.

## 2. Results

We obtained 550 mL of EO representing a yield of 1.22% *v/w* (volume/100 g of fresh material). In Table 1 is shown the composition of the EO extracted. The *M. armillaris* EO isolated for this work presented a liquid consistency with a pale-yellow color and a penetrating and fresh odor. The other parameters analyzed were the refractive index; density; pH; solubility in mineral oil, ethanol 70%, and water; acid value; and esterification index (Table 2). 

For the MIC determination assay, erythromycin was used for the quality check of the microdilution method. The MIC of this antibiotic was 0.5 μg/mL for *S. aureus* ATCC 29213 at pH 7.4 (it must range between 0.25 and 1 μg/mL, according to the CLSI (2013) [26]). The MIC of *M. armillaris* EO necessary to inhibit *S. aureus* ATCC 29213 was 25 µL/mL at pH 7.4 and 6.5 but decreased twofold at pH 5.0. Concerning wild-type strains, the MIC was 12.5 µL/mL at pH 7.4 for all strains, without a change at pH 6.5 for three strains (SA13, SA96, and SA139) and decreasing by half for the other strains at pH 6.5 (SA78A, SA79A, and SA86B). Variations may be due to the existence of bacterial subpopulations with different sensitivities. At pH 5.0, the MIC decreased by half from that obtained at pH 6.5 (6.25 and 3.1 µL/mL, respectively). Something similar occurred when evaluating the MBC, since this parameter decreased between 2 and 4 times depending on the strain, comparing what happened at pH 7.4 and 5.0. The inhibitory and bactericidal concentrations of EO against the reference strain and the six wild-type strains are shown in Table 3. 

**Figure 1:**
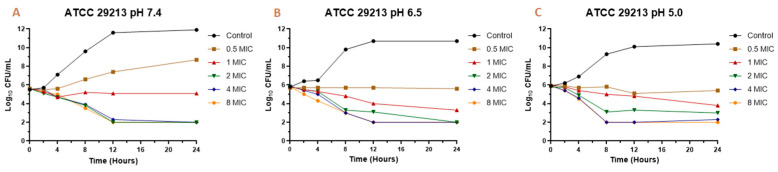
Time-kill curve for *Melaleuca armillaris* essential oil against *Staphylococcus aureus* ATCC 29213 at pH 7.4 (**A**), 6.5 (**B**), and 5.0 (**C**).

**Figure 2:**
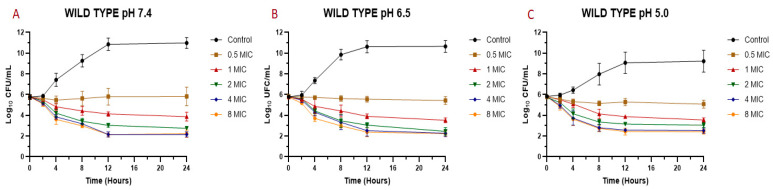
Time-kill curve for *M. armillaris* essential oil against *S. aureus* wild types (*n* = 6, using the mean of triplicates for each strain) at pH 7.4 (**A**), 6.5 (**B**), and 5.0 (**C**).

The MIC values mentioned before (Table 3) were used to perform the time-kill assay. Each strain was exposed to different concentrations of EO (0.5 MIC, 1 MIC, 2 MIC, 4 MIC, 8 MIC, and a control without EO). Figure 1 shows the time-kill assay for the reference strain and Figure 2 the wild types. In both cases, we evaluated *S. aureus* behavior at pH 7.4, 6.5, and 5.0 in the presence and absence of EO.

The bacterial growth curves were modeled with the Gompertz equation. The data obtained are presented in Table 4 (reference strain) and Table 5 (wild-type strains). At pH 6.5 and 5.0, there was a decrease in the bacterial count for 0.5 MIC; therefore, it was not possible to analyze them with this model.

Those curves where the bacterial inoculum decreased were analyzed with the sigmoid model minus the base, and the parameters obtained are observed in Table 6 (reference strain) and Table 7 (wild-type strains).

**Figure 3:**
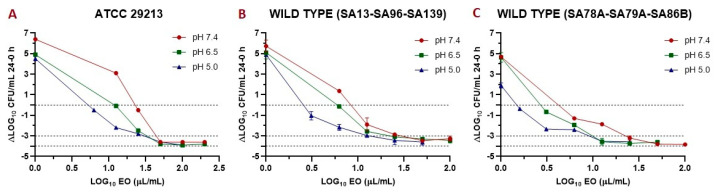
Graphic representation of the antibacterial effect (antibacterial effect (E): ΔLog colony-forming units (CFU)/mL 24–0 h) of essential oil (EO) against *S. aureus* ATCC 29213 (**A**); wild type (*n* = 3: SA13, SA96, and SA139, using the mean of triplicates for each strain) (**B**); and wild type (*n* = 3: SA78A, SA79A, and SA86B, using the mean of triplicates for each strain) (**C**). In the three cases are plotted the E vs. EO concentration at pH 7.4, 6.5, and 5.0.

The data obtained in the time-kill assay allowed us to obtain index E (antibacterial effect) of the antibacterial activity. In Figure 3, index E vs. the EO concentration is plotted at the three different pH evaluated. In this way, it is possible to observe the incidence of pH in the drop of the bacterial count added to the effect of the EO. Table 8 shows the parameters obtained after modeling this data with the sigmoid model.

## 3. Discussion

Physicochemical characterization is important to assess the quality of essential oils. It is very important for the standardization and design of commercial products, especially if they are destined for food and healthcare in both animal and human medicines. There is no information in the literature to compare the physicochemical parameters we obtained. These represent a starting point for the standardization of *M. armillaris* EO and consider them for quality control. The chromatographic analysis of this EO revealed the presence of 1.8 cineol as the main component (72.3%) and limonene (7.8%) and α-pinene (6.0%). These are commonly present in essential oils with high antimicrobial activity. The 1.8 cineole is a monocyclic monoterpene with an important antimicrobial activity and was found as the main component in *M. armillaris* EO in other works [5,6,7]. This compound is, in general, also the major compound in the essential oil of Eucalyptus species [27].

Falci et al. (2015) studied the composition and the antimicrobial activity of the essential oil of a *Melaleuca* species (not specified) cultivated in Brazil [28]. This essential oil had 70.8% of 1.8 cineole, 8.95% of terpineol, and 8.25% of limonene. The amount of 1.8 cineole, limonene, and myrcene (1.99%) was similar to the essential oil of *M. armillaris* obtained in this work. Although the specie of *Melaleuca* is not specified, parallelism can be made with the composition of the mentioned essential oil. These authors demonstrated an important antimicrobial activity against *S. aureus* strains with MIC values between 1 and 2 µL/mL and MBC between 2 and 4 µL/mL. Li et al. (2014) found that the MIC of 1.8 cineol against *S. aureus* ATCC 25923 was 6.25 μL/mL [29]. Those value reported were lower than the obtained in the present study. This can be attributed to a greater sensitivity of the *S. aureus* strains used by the authors and/or incidences in the antimicrobial activity of the rest of the minority components.

The high content of 1.8 cineol may be one of the factors that contribute to the antibacterial activity of the EO, to which the permeabilization of the membranes of microorganisms such as *S. aureus* has been attributed as an antimicrobial action mechanism due to its great hydrophobicity [30,31]. This compound is usually the most abundant in *Eucalyptus globulus* essential oil. Yáñez Rueda and Cuadro Mogollón (2012) found an important activity for this species against *S. aureus* ATCC 29213 (MIC of 12.4 μg/mL), in which its composition was similar to the *M. armillaris* EO evaluated in this work: 1.8 cineol (82.27%), limonene (3.70%), α-pinene (3.16%), terpinen-4-ol (1.4%), α-terpineol (1.2%), β-myrcene (1.12%), and α-terpinene (1.1%), among others [32]. This could indicate a synergism between these components particularly effective against strains of *S. aureus*.

According to the ratio of MIC/MBC, an antimicrobial may be considered bactericidal or bacteriostatic. A compound is bacteriostatic if the MBC/MIC ratio is greater than 4 [33]. Analyzing the MIC and MBC of the EO, we found that, for strains SA13, SA96, and SA139, these parameters were the same, and this coincidence was maintained even when the pH conditions were modified. For the other strains, the MBC/MIC ratio was between 2 and 4, maintaining the ratio when acidifying the culture medium. Therefore, it could be considered that the EO of *M. armillaris* has bactericidal activity against *S. aureus*, which is independent of the pH.

The *M. armillaris* EO mechanism of action has not yet been investigated against *S. aureus*. Hayouni et al. (2008) studied the antimicrobial activity of this species against different *Lactobacillus* species [6]. As 1.8 cineole was the main component found (68.92%), these authors hypothesized that this compound could have destabilized the cytoplasmic membrane of these bacteria, as was demonstrated by Li et al. (2014) [29]. However, the way of action postulated for *M. armillaris* by Hayouni et al. (2008) also involved the minority components found (α-pinene, terpinen-4-ol, sabinene, β-myrcene, and α-terpinene, among others) [6]. According to these authors, these molecules interact with the cell membrane, where they dissolve in the phospholipid bilayer, aligning themselves between the fatty acid chains. This distortion of the physical structure would cause the expansion and destabilization of the membrane, increasing the fluidity of the membrane, which, in turn, would increase the passive permeability.

The MIC and MBC are the parameters most used to quantify the antibacterial activity of a drug against an infectious pathogen. However, the temporal evaluation of different concentrations of the antimicrobial against a microorganism allows a better description of the magnitude of its antibacterial effect [34]. For this reason, it is also important to analyze what occurs over time through the construction of bacterial death curves. In the time-kill assay for the EO against *S. aureus* (Figure 1 and Figure 2), it is possible to observe a decrease in the bacterial count after being exposed to the EO. In general, a slight drop in the slope of the curve was observed with a concentration equivalent to the MIC of the essential oil against each strain and isolate. However, for concentrations of 0.5 MIC, a relevant rate of growth was not perceived, and in many cases, there was a decrease in the initial inoculum. For concentrations of two, four, and eight times the MIC, a drop in the bacterial cell count was evidenced at two hours, continuing the decrease exponentially until 8–12 h and then maintaining the bacterial count until 24 h after assay started. This pattern was generally maintained for all strains, even changing the pH of the medium.

In the case of the reference strain (ATCC 29213) at pH 7.4, it was possible to achieve a decrease of 2.6 Log_10_ (colony-forming units (CFU)/mL) of the initial inoculum for concentrations of two, four, and eight times the MIC. At pH 6.5, the decrease for these concentrations was 2.8 Log_10_ (CFU/mL). At pH 5.0, the decrease in inoculum was 2.8 for two MIC, 3.6 for four MIC, and 3.9 Log_10_ (CFU/mL) for eight MIC. With these results, we can observe that, at higher concentrations and higher acidity, the antibacterial activity of the essential oil is higher, being similar for concentrations of four and eight MIC. For strains SA13, SA96, and SA139, the decrease in the initial inoculum for two MIC at 24 h is 2.7–3.0 Log_10_ (CFU/mL) at pH 7.4, 3.0–3.2 at pH 6.5, and 2.9–3.2 at pH 5.0. As for four and eight MIC, the decrease is 2.5–3.5 Log_10_ (CFU/mL) at pH 7.4, from 3.1–3.7 at pH 6.5, and 3.2–3.9 at pH 5.0. Regarding strains SA78A, SA79A, and SA86B, a fall of 3.1–3.8 Log_10_ (CFU/mL) was observed for the three pH values at concentrations of four and eight MIC. In the case of the two MIC for these strains, the drop in inoculum was between 2.5 and 3.4 Log_10_ (CFU/mL). The strains analyzed had slight differences in susceptibility against the EO but the antimicrobial activity improvement by increasing the acidity and EO concentration was common.

The mathematical modeling of a microorganism response at different conditions or with an inhibitor compound is very useful to understand its behavior and to predict the efficacy of a treatment under controlled conditions. To assess the validity of the model applied, the model must have a good fit to experimental data in terms of R^2^, which must range between 0 and 1; the adjustment is better if this parameter is nearer to 1 [22].

The application of the Gompertz model to data obtained from the time-kill assay allows us to know parameters like the growth rate (μ), lag period duration (LPD), and the maximum population density (MPD). We used this model for the bacterial growth being applied for the control conditions and 0.5 MIC at pH 7.4 (Table 4 and Table 5). With these results, we could observe that the presence of EO diminished the μ, extended the LPD, and reduced the MPD. Something similar occurred in another study using *M. armillaris* EO against lactic acid bacteria [6]. These parameters also changed in a same way because of the pH decrease, highlighting the lower growth capacity of *S. aureus* under acidic conditions. Weinrick et al. (2004) found that *S. aureus* in acidic conditions modifies its gene expression to promote defense mechanisms against acidity, which can lead to a slower growth rate [35].

At pH 6.5 and 5.0, only the control increased the bacterial count, so the Gompertz model was not applied for 0.5 MIC. In this case, and for curves obtained using one MIC, two MIC, four MIC, and eight MIC (where bacterial death was observed), we applied the sigmoid minus the base model (Table 6 and Table 7). With this model, we obtained the T_I50_ (time to reach 50% of the maximum drop in the bacterial count, N_max_). This parameter is lower while the EO concentration increases, and the reduction is independent of the pH. On the other hand, N_max_ is much closer to N_0_ with a higher concentration of EO, indicating that there is a much greater bacterial effect, since it is possible to eliminate all the initial inoculum. Navarro-Cruz et al. (2018) found that, when modeling the antibacterial effect of the essential oil of *Lippia berlandieri* against *S. aureus*, the time needed to decrease the initial inoculum by 50% was shorter when modifying the pH from 7 to 5 [36], coinciding with our findings for *M. armillaris*.

The antibacterial effect (Index E) of EO is shown in Figure 3. The EO improves its antimicrobial activity at a lower pH, since lower concentrations are required to achieve the same effect. This behavior was similar for both the reference and wild-type strains. Modeling this data by applying a sigmoid model let us obtain different valuable parameters (Table 8), such as the concentration necessary to reach 50% of the maximum effect (C_50_). This parameter was smaller at lower pH for all the strains evaluated. Another important parameter is the E_max_; this decreases at acidic pH, but we must consider that the E_0_ is also smaller; this is because *S. aureus* is slightly susceptible at acidic pH. The lower maximum antibacterial effect at a lower pH may be influenced by a lower bacterial growth capacity, coinciding with that demonstrated by Weinrick et al. (2004) [35]. At high concentrations, the antibacterial effect was similar (and close to virtual eradication) for the three pH, while at acidic pH, the E_0_ was lower, which affected the value of the E_max_.

## 4. Materials and Methods

### 4.1. M. armillaris Essential Oil Extraction

The collection of leaves and herbaceous branches was carried out in Coronel Brandsen, Buenos Aires, Argentina (latitude 35°06’18.9” S and longitude 58°10´57.0” W). A sample portion was reserved for identification and further storage at the LPAG herbarium at the Faculty of Agrarian and Forestry Sciences, UNLP [37]. EO was obtained by steam distillation of the whole collected fresh biomass (44.85 kg). Subsequently, the EO was dried with sodium sulfate anhydrous at room temperature, filtered with a cotton funnel, and stored at 4 °C in an amber glass bottle.

### 4.2. Essential Oil Characterization

The EO composition was analyzed by GC–MS-FID, as we previously described [4]. We performed assays on the established parameters of quality control commonly used for essential oils [38] to characterize the EO of *M. armillaris*, since it is not described in the literature. In this way, the physicochemical characteristics such as appearance at 20 °C, odor, flavor, color, refractive index, density (using a pycnometer), and pH were analyzed. Additionally, we checked the solubility in different solvents: mineral oil (1:1); water (1:10), and ethanol 70% (1:1). On the other hand, the acid value and esterification index were determined following the recommendations of Argentinian Pharmacopeia VII Ed (2013) [39]:

Acid value (amount of free fatty acids, defined as the number of mg of potassium hydroxide necessary to neutralize the free acids present in 1.0 g of sample): 10.0 g of sample, exactly weighed and previously neutralized against phenolphthalein with 0.1-N sodium hydroxide, were dissolved in 50 mL of alcohol contained in an Erlenmeyer flask (Becton Dickinson^®^). One milliliter of phenolphthalein (prepared at 1% in alcohol) was added, and it was titrated with 0.1-N potassium hydroxide until a persistent pink coloration for 30 s. The acid number was calculated as the mg of KOH necessary to neutralize the free fatty acids in one gram of sample. All reagents were purchased from Sigma Aldrich, St. Louis, MO, USA.

Esterification index (defined as the number of mg of potassium hydroxide necessary to saponify the esters present in 1.0 g of sample): 2 g of sample, exactly weighed, was transferred to a 250-mL Erlenmeyer, previously weighed, and 25 mL of neutralized alcohol were added while stirring, and 1 mL of phenolphthalein (prepared in a 1% ethanol solution) was added. It was titrated with 0.5-N alcoholic potassium hydroxide until completely neutralizing the free fatty acids. Then, 25.0 mL of 0.5-N alcoholic potassium hydroxide was added. It was heated in a water bath, with an appropriate coolant to maintain reflux for 30 min, stirring frequently, and excess potassium was titrated with 0.5-N hydrochloric acid. A determination was made with a blank. The difference between the volumes, in mL, of 0.5-N hydrochloric acid consumed by the sample and the blank, multiplied by 28.05, and divided by the weight, in g, of the sample taken, is the esterification index.

### 4.3. Inhibitory and Bactericidal Activity of M. armillaris Essential Oil Against S. aureus

Six wild-type (*n* = 6) *S. aureus* isolated, according to National Mastitis Council procedure [40], from subclinical mastitis Holstein cows were used. The protocol followed the Guide for the Care and Use of Agricultural Animals in Agricultural Research and Teaching (Federation of Animal Science Societies—FASS) and was approved by the Institutional Committee (CICUAL) of the Faculty of Veterinary Sciences, National University of La Plata, Buenos Aires, Argentina (47.3.15J). The strains were identified phenotypically as a Gram-positive, catalase-positive, β-hemolytic, Voges Proskauer-positive fermentation of trehalose, mannitol, and maltose. The isolates are part of the strains collection of our Laboratory (Laboratorio de Estudios Farmacológicos y Toxicológicos -LEFyT- Faculty of Veterinary Sciences, UNLP). Minimum inhibitory concentration (MIC) and minimum bactericidal concentration (MBC) of EO were established by broth microdilution method in 96-well polystyrene microplates (Becton Dickinson^®^). Mueller Hinton Broth (MHB) culture medium was used with the addition of 0.5% of Tween 80. This surfactant enhanced the dissolution of the EO in the aqueous culture medium. MICs and MBCs were evaluated at 3 different pH to emulate extracellular and intracellular (inside cytosol or phagolysosome) conditions where *S. aureus* was internalized (7.4, 6.5, and 5.0, respectively). This pH adjustment of the medium was carried out by addition of HCl to the broth. The range of essential oil concentrations evaluated was 50 to 0.09 µL/mL, and each plate was inoculated with a final bacterial concentration of 5 × 10^5^ CFU/mL and incubated at 35 °C for 18–24 h. MIC was established as the lowest concentration inhibiting bacterial growth. Positive (without antimicrobials) and negative controls (without antimicrobials and inoculums) with MHB containing 0.5% Tween 80 were performed. Every determination for each strain was evaluated at the different 3 pH conditions by triplicate. For quality control, *S. aureus* ATCC 29213^®^ was used, and the susceptibility to erythromycin was checked for this strain by the control procedure [26].

Once the MIC was established, 25 μL were taken from each well, showing no evident bacterial growth, then inoculated individually in nutritive agar plates for colony counting after incubation at 35 °C for 18–24 h. MBC was the lowest antimicrobial concentration in which the initial inoculum fell (99.9%).

### 4.4. Time-kill Assay and Antibacterial Activity Index of the EO

Once the MICs of the EO and its combinations were identified, data were used to perform time-kill assays to evaluate the antibacterial activity index (E). Each *S. aureus* strains were faced with different concentrations (0.5 MIC, 1 MIC, 2 MIC, 4 MIC, and 8 MIC) of EO, including the quality control strain *S. aureus* ATCC 29213^®^.

We prepared 7 tubes, one for each concentration, and a positive (without antimicrobials) and a negative (without antimicrobials and inoculums) control. Each one contained a final volume of 1 mL including MHB with 0.5% Tween 80 (pH 7.4, 6.5, and 5.0), antimicrobials, and a final inoculum of 5 × 10^5^ CFU/mL. Incubations were carried out at 35 °C. Bacterial plate count was carried out at the initial time, 2, 4, 8, 12, and 24 h after incubating at 35 °C by 24 h. The assay was performed in triplicate for each strain.

Data obtained in the time-kill assay was used to create CFU/mL vs. time graphs and to evaluate the antibacterial activity index (E). Once E indexes were obtained, we graphed the E index vs. EO concentration (Log_10_) to compare what happens at the 3 pH evaluated. The wild strains were grouped according to the MIC, obtaining two groups of three strains for each one (using the mean of triplicates for each strain). Graphics were plotted using the GraphPad Prism 6 program (GraphPad Software, Inc).

E index was defined as the difference in Log_10_ between the bacterial count (CFU/mL) at the initial time (nt-0) and at the end of the test (nt-24): E = (nt-24) − (nt-0). We considered 3 theoretical breakpoints to establish the bacteriostatic effect (E = 0), bactericidal effect (E = −3), and effect of virtual eradication of bacteria (E = −4) [41].

### 4.5. Modeling Bacterial Growth and Death for S. aureus in Presence of EO

With data obtained in the time-kill assay, we carried out the mathematical modeling to describe the growth and death of *S. aureus* in presence of EO. For growth, we applied the Gompertz model obtaining the specific growth rate (μ), the lag phase duration (LPD), and the maximum population density (MPD) [23]. The mathematical expression of this model is:

Log N = a + c. exp (−exp (−b (t − m)))(1)

where Log N is the decimal logarithm of the microbial counts (Log_10_ CFU/mL) at time t (hours), a is the Log_10_ of the asymptotic bacterial counts when the time decreases indefinitely (Log_10_ CFU/mL), c is the Log_10_ of the asymptotic counts when the time increases indefinitely (Log_10_ CFU/mL), m is the time required to reach the maximum growth rate (hours), and b is the growth rate relative to time m (hours^−1^). Therefore, we can obtain: µ = b. c/e (Log_10_ CFU/mL. hours), where e = 2.7182, LPD = m -1/b (hours), and MPD = a + c (Log_10_ CFU/mL). The equation was fitted to the microbial development data using a nonlinear regression with the Sigma Plot program (Sigma Plot 12.0, 2011), since the parameters of the Gompertz model are nonlinear.

In the case of the curves where bacterial death was observed, the experimental data of CFU/mL vs. time were fitted with a sigmoid model minus the base:

N = N_0_ − (N_max_. T^γ^)/(T_I50_^γ^ + T^γ^) (2)

where N is the bacterial count (CFU/mL) at time T (hours), N_0_ is the initial inoculum concentration (CFU/mL), N_max_ is the maximum drop in the bacterial count (CFU/mL), T_I50_ is the time necessary to reach 50% of the maximum bacterial inhibition (hours), and ϒ is the sigmoidicity coefficient. Experimental data were fitted with the nonlinear least squares regression model using the software Sigma Plot 12.0, as mentioned before.

Finally, we applied the sigmoid model, which is analogous to the maximum response, or Hill [25,42] to the values of index E vs. EO concentration to understand the mechanics of the relationship between the concentration of these and their bactericidal effects and, thus, be able to obtain more information about the behavior of *S. aureus* under the different conditions evaluated. Redefining the previous equation:

E = E_0_ − (E_max_. C^γ^)/(C_50_^γ^ + C^γ^) (3)

where E is the index E (Log_10_ CFU/mL) for a concentration C (μL/mL), E_0_ is the index E in the absence of the antimicrobial (Log_10_ CFU/mL), E_max_ is the maximum reduction in Log_10_ of E_0_, C_50_ (μL/mL) is the concentration that causes 50% of the reduction of the E_max_, and ϒ is the coefficient of sigmoidicity. The experimental data were fitted with the nonlinear least squares regression model using Sigma Plot software (Sigma Plot 12.0, 2011).

## 5. Conclusions

The essential oil of *M. armillaris* has good antimicrobial activity against *S. aureus*. This improves slightly with the acidification of the culture medium and presents a bactericidal activity where the MBC is close to the MIC. The analysis of biological systems using mathematical models allows to obtain more information that simplifies collecting data from the observation of the results of an in vitro test. We highlighted the antimicrobial potential of *M. armillaris* EO against *S. aureus* under acidic conditions, resulting in an interesting factor for the control of *S. aureus* infections and food contamination.

## Figures and Tables

**Table 1:** Composition of *Melaleuca armillaris* essential oil analyzed by gas chromatography-mass spectrometry and flame ionization detector (GC-MS)-FID. RI: retention index. The polar column was connected to an FID, whereas the nonpolar column was connected to a MS detector.

Compound	RI Nonpolar	RI Polar	AREA %
1,8-Cineole	1022	1234	72.3
Limonene	1024	1221	7.8
α-Pinene	935	1043	6.0
Myrcene	974	1170	2.2
β-Pinene	979	1133	2.2
α-Thujene	926	1036	1.5
p-Cymene	1018	1286	1.4
Terpinen-4-ol	1164	1614	1.4
α-Terpineol	1172	1705	1.4
Sabinene	968	1138	1.0
γ-Terpinene	1047	1264	0.5
β-Caryophyllene	1417	1614	0.5
α-Terpinene	1012	1206	0.2
trans-β-Ocimene	1032	1260	0.2
Geranyl acetate	1359	1760	0.2
α-Phellandrene	1005	1191	0.1
Terpinolene	1082	1305	0.1
δ-Terpineol	1150	1674	0.1
Aromandendrene	1437	1622	0.1
Geranyl isobutyrate	1496	1794	0.1
cis-Calamenene	1508	1841	0.1
Oxi-Caryophyllene	1565	1989	0.1

**Table 2:** Physicochemical parameters of *M. armillaris* essential oil.

Parameter	Value Obtained
Appearance at 20 °C	Oily and transparent liquid
Odor	Penetrating, very fresh
Taste	Bitter, astringent
Color	Pale-yellow
Density (using pycnometer)	0.89197–0.93013 g/mL
Solubility in mineral oil (1:1)	Soluble
Solubility in water (1:10)	Partially soluble
Solubility in ethanol 70% (1:1)	Soluble
Refractive index at 20 °C	1.4698–1.4703
Acid value	0.7824
Esterification index	32.8526

**Table 3:** The MIC (minimum inhibitory concentration) and MBC (minimum bactericidal concentration) in μL/mL of each strain analyzed at pH 7.4, 6.5, and 5.0.

Strain	pH 7.4		pH 6.5		pH 5.0	
	MIC	MBC	MIC	MBC	MIC	MBC
ATCC 29213	25	50	25	50	12.5	25
SA 13	12.5	25	12.5	25	6.25	25
SA 96	12.5	50	12.5	25	6.25	12.5
SA 139	12.5	25	12.5	25	6.25	12.5
SA 78A	12.5	12.5	6.25	6.25	3.1	3.1
SA 79A	12.5	12.5	6.25	6.25	3.1	3.1
SA 86B	12.5	12.5	6.25	6.25	3.1	3.1

**Table 4:** Parameters estimated by the Gompertz model for the ATCC 29213 strain at pH 7.4, 6.5, and 5.0.

Parameter	pH 7.4		pH 6.5		pH 5.0	
	Control	0.5 MIC	Control	0.5 MIC	Control	0.5 MIC
R^2^	0.988	0.908	0.996	-	1.000	-
µ (Log_10_ CFU/mL*hours)	0.74	0.72	0.70	-	0.67	-
LPD (hours)	1.80	9.96	2.48	-	2.54	-
MPD (Log_10_ CFU/mL)	12.20	8.66	10.84	-	10.40	-

R^2^: coefficient of determination, μ: growth rate, LPD: latency period duration, MPD: maximum population density, and CFU: colony-forming units.

**Table 5:** Mean of the parameters estimated by the Gompertz model for the wild-type strains at pH 7.4, 6.5, and 5.0.

Parameter	pH 7.4		pH 6.5		pH 5.0	
	Control	0.5 MIC*	Control	0.5 MIC	Control	0.5 MIC
R^2^	0.990	0.972	0.994	-	0.992	-
µ (Log_10_ CFU/mL*hours)	0.72	0.52	0.71	-	0.49	-
LPD (hours)	1.86	6.81	1.93	-	2.92	-
MPD (Log_10_ CFU/mL)	12.06	6.86	10.91	-	9.27	-

R^2^: coefficient of determination, μ: growth rate, LPD: latency period duration, and MPD: maximum population density. The 0.5 MIC* strain 78A was excluded, because it could not be analyzed by Gompertz.

**Table 6:** Parameters estimated by the sigmoid model minus the base for the ATCC 29213 strain at pH 7.4, 6.5, and 5.0.

	Parameter	0.5 MIC	1 MIC	2 MIC	4 MIC	8 MIC
pH 7.4	R^2^	-	0.925	1.000	1.000	1.000
	N_max_ (CFU/mL)	-	297,000	375,000	371,000	420,000
	ϒ	-	29.33	1.91	1.70	1.92
	T_I50_ (hours)	-	1.96	1.53	1.36	1.36
	N_0_ (CFU/mL)	-	410,000	370,000	365,000	415,000
pH 6.5	R^2^	0.949	0.998	0.990	0.998	1.000
	N_max_ (CFU/mL)	162,000	679,000	716,000	786,000	591,000
	ϒ	5.62	1.48	1.49	1.85	2.68
	T_I50_ (hours)	11.53	2.30	1.94	1.54	1.16
	N_0_ (CFU/mL)	540,000	649,000	679,000	770,000	59,000
pH 5.0	R^2^	0.776	0.998	1.000	1.000	1.000
	N_max_	782,000	897,000	680,000	782,000	821,000
	ϒ	1.30	2.48	4.60	3.31	3.35
	T_I50_ (hours)	6.40	2.82	2.54	1.68	1.59
	N_0_ (CFU/mL)	877,000	922,000	680,000	780,000	820,000

R^2^ is the coefficient of determination, N_0_ is the initial inoculum concentration, N_max_ is the maximum drop in the bacterial count, T_I50_ is the time necessary to reach 50% of the maximum bacterial inhibition, and ϒ is the sigmoidicity coefficient.

**Table 7:** Mean of the parameters estimated by the sigmoid minus the base model for the wild-type strains at pH 7.4, 6.5, and 5.0.

	Parameter	0.5 MIC	1 MIC	2 MIC	4 MIC	8 MIC
pH 7.4	R^2^	-	0.968	0.994	0.998	1.000
	N_max_ (CFU/mL)	-	714,000	649,000	657,000	646,000
	ϒ	-	17.16	3.93	4.02	4.32
	T_I50_ (hours)	-	7.62	2.38	2.23	1.87
	N_0_ (CFU/mL)	-	673,000	702,000	654,000	640,000
pH 6.5	R^2^	0.943	0.996	1.000	1.000	1.000
	N_max_ (CFU/mL)	322,000	633,000	640,000	651,000	595,000
	ϒ	23.58	9.67	6.02	4.94	6469.42
	T_I50_ (hours)	5.62	2.83	2.28	1.95	1.65
	N_0_ (CFU/mL)	671,000	604,000	643,000	653,000	595,000
pH 5.0	R^2^	0.962	0.998	1.000	1.000	1.000
	N_max_ (CFU/mL)	490,000	667,000	564,000	646,000	634,000
	ϒ	6.85	3.44	4.46	4.52	4.50
	T_I50_ (hours)	3.25	2.37	1.71	1.34	1.28
	N_0_ (CFU/mL)	631,000	669,000	563,000	645,000	633,000

R^2^ is the coefficient of determination, N_0_ is the initial inoculum concentration, N_max_ is the maximum drop in the bacterial count, T_I50_ is the time necessary to reach 50% of the maximum bacterial inhibition, and ϒ is the sigmoidicity coefficient.

**Table 8:** Parameters obtained by the sigmoid model applied to curves of the Index E (antibacterial effect) (CFU/mL) vs. EO concentration (μL/mL) at pH 7.4, 6.5, and 5.0.

Strain	Parameter	pH 7.4	pH 6.5	pH 5.0
ATCC 29213	R^2^	0.996	0.998	0.998
	E_max_ (Log_10_ CFU/mL)	10.20	8.85	8.63
	ϒ	2.42	2.14	1.12
	C_50_ (μL/mL)	17.56	11.13	4.56
	E_0_ (Log_10_ CFU/mL)	6.36	4.90	4.50
WT (13–96-139)	R^2^	0.998	1.000	0.998
	E_max_ (Log_10_ CFU/mL)	9.11	8.46	8.85
	ϒ	2.71	2.51	1.01
	C_50_ (μL/mL)	6.52	5.15	1.50
	E_0_ (Log_10_ CFU/mL)	5.73	5.10	4.90
WT (78A-79A-86B)	R^2^	0.996	0.994	0.978
	E_max_ (Log_10_ CFU/mL)	9.35	8.56	5.48
	ϒ	0.75	1.41	1.68
	C_50_ (μL/mL)	3.13	2.28	1.86
	E_0_ (Log_10_ CFU/mL)	4.70	4.63	1.92

R^2^ is the coefficient of determination, E_0_ is the index E (antibacterial index) in the absence of an antimicrobial, E_max_ is the maximum reduction in Log_10_ of E_0_, C_50_ is the concentration that causes 50% of the reduction of the E_max_, ϒ is the coefficient of sigmoidicity, and WT stands for wild type.

## Data Availability

The data presented in this study are available on request from the corresponding author.
